# Prevalence of intimate partner violence against women in the Arab world: a systematic review

**DOI:** 10.1186/s12914-019-0215-5

**Published:** 2019-10-22

**Authors:** Tatiana Elghossain, Sarah Bott, Chaza Akik, Carla Makhlouf Obermeyer

**Affiliations:** 1 Center for Research on Population and Health at the Faculty of Health Sciences, American University of Beirut, Beirut, Lebanon; 2 Independent researcher, consultant to the Center for Research on Population and Health at the Faculty of Health Sciences, American University of Beirut, Beirut, Lebanon

**Keywords:** Violence against women, Intimate partner violence, Domestic violence, Spouse abuse, Gender norms, Sexual violence, Arab region

## Abstract

**Background:**

Violence against women has particular importance for women’s health and wellbeing in the Arab world, where women face persistent barriers to social, political and economic equality. This review aims to summarize what is known about the prevalence of physical, sexual and emotional/psychological intimate partner violence (IPV) against women in the 22 countries of the Arab League, including geographic coverage, quality and comparability of the evidence.

**Methods:**

A systematic review of IPV prevalence in Arab countries was carried out among peer-reviewed journal articles and national, population-based survey reports published by international research programmes and/or governments. Following PRISMA guidelines, Medline and the Social Sciences Citation Index were searched with Medical Subject Headings terms and key words related to IPV and the names of Arab countries. Eligible sources were published between January 2000 and January 2016, in any language. United Nations databases and similar sources were searched for national surveys. Study characteristics, operational definitions and prevalence data were extracted into a database using Open Data Kit Software. Risk of bias was assessed with a structured checklist.

**Results:**

The search identified 74 records with population or facility-based IPV prevalence data from eleven Arab countries, based on 56 individual datasets. These included 46 separate survey datasets from peer-reviewed journals and 11 national surveys published by international research programmes and/or governments. Seven countries had national, population-based IPV estimates. Reported IPV prevalence (ever) ranged from 6% to more than half (59%) (physical); from 3 to 40% (sexual); and from 5 to 91% (emotional/ psychological). Methods and operational definitions of violence varied widely, especially for emotional/psychological IPV, limiting comparability.

**Conclusions:**

IPV against women in Arab countries represents a public health and human rights problem, with substantial levels of physical, sexual and emotional/psychological IPV documented in many settings. The evidence base is fragmented, however, suggesting a need for more comparable, high quality research on IPV in the region and greater adherence to international scientific and ethical guidelines. There is a particular need for national, population-based data to inform prevention and responses to violence against women, and to help Arab countries monitor progress towards the Sustainable Development Goals.

## Background

Despite substantial socio-economic diversity, the 22 countries^1^ of the Arab League [1] (also referred to as the Arab region) share commonalities in language (Arabic), culture and religion (majority Islam). Some indicators of women’s status across the region have improved in recent years. For example, almost all Arab countries have achieved gender parity in primary and secondary education (with exceptions such as Morocco and Yemen), a dramatic change from parity rates of 40–50% in the 1960s; and women’s university enrolment exceeds that of men in Algeria, Bahrain, Jordan, Kuwait, Lebanon, Qatar, Oman and Saudi Arabia [2]. Nonetheless, by many measures, Arab women across the region continue to face barriers to full social, economic and political equality. All 14 Arab countries included in the 2017 World Economic Forum Global Gender Gap index ranked in the bottom 28 of 144 countries, based on gender gaps in health, education, political empowerment and economic participation [3]. A 2013 analysis found that women’s labour participation was the lowest of all regions — 26% compared with a world average of 51% [2]. While all Arab countries, apart from Somalia and Sudan, have signed the Convention on the Elimination of All forms of Discrimination Against Women, many did so with reservations about gender discrimination; equal rights in marriage, family, employment or nationality; right to choose residence; and/or compatibility with religious laws [4]. While female age at marriage has risen dramatically in some high-income Arab states, rates of child marriage remain substantial in countries such as Mauritania, Somalia, Sudan, Yemen, parts of Egypt, and among Palestinians in Gaza [5].

A recent United Nations (UN) analysis highlighted links between violence against women (VAW) in Arab countries and societal factors such as lack of female political and economic participation, discriminatory legal codes, legal impunity for violence against women and girls, and – in some settings – armed conflict and forced displacement [4]. Studies also suggest that rigid gender norms and notions of masculinity embedded in traditional culture contribute to men’s use of VAW [6]. Polarizing debates about women’s status in the Arab region – between those who argue that traditional interpretations of Islamic doctrine protect women and those who criticize social norms supporting male control over women – make it all the more important to understand levels of VAW in the region [4]. Intimate partner violence (IPV), the most common form of VAW, is widely recognized as a human rights problem that poses a significant threat to women’s health and wellbeing – both globally [7] and within the Arab region [4]. In 2013, the World Health Organization (WHO) estimated that 30% of ever-partnered women worldwide had experienced physical or sexual violence by a partner; as had 37% of ever-partnered women in the Eastern Mediterranean region, which covers part of the Arab region [7]. Other than South East Asia, this was the highest estimate of any region, though it was based on data from just four Arab countries (Egypt, Iraq, Jordan and Palestine) plus Iran.

All 22 Arab countries agreed to the UN Sustainable Development Goals (SDGs), including Target 5.2: Eliminate all forms of violence against all women and girls in public and private spheres [8]. They also agreed to measure SDG indicators, such as 5.2.1: The proportion of ever-partnered women and girls aged 15 years and older subjected to physical, sexual or psychological violence by a current or former intimate partner. IPV prevalence data from the Arab region are limited, however. As of July 2019, the SDG global databases contained national estimates for Indicator 5.2.1 from only three Arab countries (those with a DHS violence module, Comoros, Egypt and Jordan) [9]. A 2017 UN review identified 14 studies with IPV prevalence from six Arab countries [4], but there has not been a comprehensive review of IPV from the region. To address that gap, this systematic review aims to describe what is known about the prevalence of IPV against women in the 22 countries of the Arab League, including the geographic coverage, quality and comparability of the evidence.

## Methods

### Definitions and forms of IPV

The SDG metadata define IPV as any physical, sexual or emotional/psychological abuse “perpetrated by a current or former partner within the context of marriage, cohabitation or any other formal or informal union” [10]. In practice, however, researchers classify, define and measure IPV in diverse ways. Because prevalence rates in this review came from published sources, we did not try to impose standardized definitions. Instead, operational definitions were extracted from each source (when available) and described as part of the findings. Most sources defined an ‘intimate partner’ as a husband, but a few included cohabiting partners; this article uses ‘intimate partner’ and ‘partner’ interchangeably. For ease of communication, the term ‘emotional/psychological’ is used to refer to all forms of violence that researchers variously labelled as ‘emotional’, ‘psychological’, ‘verbal’, ‘social’ or ‘threat’ abuse, and in some cases controlling behaviour. To limit the scope, data on ‘controlling behaviour’ was not extracted if researchers considered it a separate domain from IPV.

### Search strategy

Eligible data sources were identified, screened and summarized using a two-stage approach: (1) a systematic search for studies reported in peer-reviewed journal articles; and (2) a search for nationally-representative, population-based surveys published by governments or international research programmes such as Demographic and Health Surveys (DHS). The search did not include other grey literature.

#### Part 1: search for peer-reviewed journal articles

In accordance with PRISMA guidelines, a search for peer-reviewed journal articles in Medline and the Social Sciences Citation Index was carried out in duplicate (TE, CA), using Medical Subject Headings (MeSH) terms and key words such as: intimate partner violence, domestic violence, spouse abuse, violence against women, gender-based violence and names of each country in the Arab League, supplemented by hand searching bibliographies. Titles and abstracts were retrieved, downloaded into Endnote and screened independently for eligibility by two authors (TE, CA). Articles selected for full text review were screened by at least two authors (TE, CA, SB). Differences were resolved by consensus.

##### Inclusion criteria

Journal articles met a priori inclusion criteria if they were:

- published in a peer reviewed journal between January 2000 – January 2016, in any language (e.g. English, French, Arabic);
- from a member country of the Arab league (including populations of foreign nationals living in Arab countries, but not Arab populations living outside member countries);
- presented data gathered among women (not samples composed exclusively of children or adolescents);
- presented percentages of women who reported any physical, any sexual, any emotional/psychological IPV, or a composite measure, but not just individual acts (e.g. slapping);
- met basic reporting quality criteria, including a clear description of research design, study population, sample size and respondent selection.

To capture a wider range of studies and to assess both the strengths and weaknesses of the evidence base, we included some articles where the operational definition of IPV was not entirely clear, if they met all other inclusion criteria.

Because the principle aim of the search was to identify surveys that gathered IPV prevalence data, sources were excluded if they were editorials, case reports, correspondence, book reviews, historical or political science analyses; and systematic reviews were retrieved but not included in publication counts. Studies were also excluded if samples were composed entirely of women who reported violence; if they focused on consequences, social determinants, and attitudes but had no prevalence data; or if they focused exclusively on forms of violence other than IPV, such as conflict-related violence, ‘honour killings’, Female Genital Mutilation (FGM), or forced marriage. Secondary analyses of national surveys were excluded if they duplicated findings reported more completely in reports retrieved through the second search arm (below).

#### Part 2: search for national, population-based surveys

The second arm searched for national, population-based surveys from Arab countries using databases of UN Women [11], the SDGs [9], the Global Health Data Exchange, DHS, and Google Scholar, and bibliographies of global and regional reviews. Because national survey findings do not always reach journals in a timely way if at all, peer-reviewed journal publication was not required for this arm of the search. National surveys were included if they met all inclusion criteria of the first arm (other than journal publication) and were:

- population-based, nationally-representative household surveys;
- part of an international research program (e.g. DHS) or carried out by, or in collaboration with, a government agency such as a national statistical office;
- published a final report (at least online) with national estimates of IPV against women (not just adolescents) between January 2000–January 2016.

### Data extraction from journal articles

Open Data Kit software [12] was used to extract data from eligible articles including: a) article identification (title, authors, year of publication, journal name, country/ies); b) study characteristics (design, methods, setting, years of data collection, and sample size/characteristics; c) instruments and operational definitions of IPV and ‘partner’; d) and prevalence rates of physical, sexual and emotional/psychological IPV against women, controlling behaviours, economic abuse, and composite measures as published.

### Risk of bias assessment

Risk of bias was assessed using a checklist adapted from existing tools [13, 14], informed by international guidelines for violence research [15, 16]. Studies received a point for each quality criteria ***not*** met including: 1) national; 2) population-based (not facility-based, except for studies of violence during pregnancy); 3) sample representative of target population, not convenience; 4) adequate sample size (> 300 or based on a precision calculation); 5) breakdown of respondent characteristics; 6) response rate ≥ 70%; 7) clear operational definitions of violence and ‘partner’; 8) reasonably valid and reliable measure (e.g. modified conflict tactics scale; partner and behaviourally-specific); 9) clearly defined, sensible numerators and denominators, including timeframe; 10) dedicated violence study, not a module in a health or alcohol study; and 11) clear adherence to WHO ethical guidelines regarding privacy, consent and confidentiality [17]. The possible range of scores was 0–11, with 0 meaning the least risk of bias and 11 meaning the highest. Scoring was performed in duplicate (SB, CA) with discrepancies resolved by consensus.

### Presentation of findings

Tables present findings by dataset (i.e. individual survey), not publication. When publications reported different rates based on the same dataset (usually because they used different subsamples to explore a specific correlate), we included rates from the source reporting prevalence among the broadest sample of women. Data from population-based studies are presented separately from (health, religious and educational) facility-based studies because the latter may not be representative of the broader population of women. IPV prevalence rates in pregnancy are presented in a dedicated table.

## Results

### Eligible sources by type and geographic coverage

**Fig. 1:**
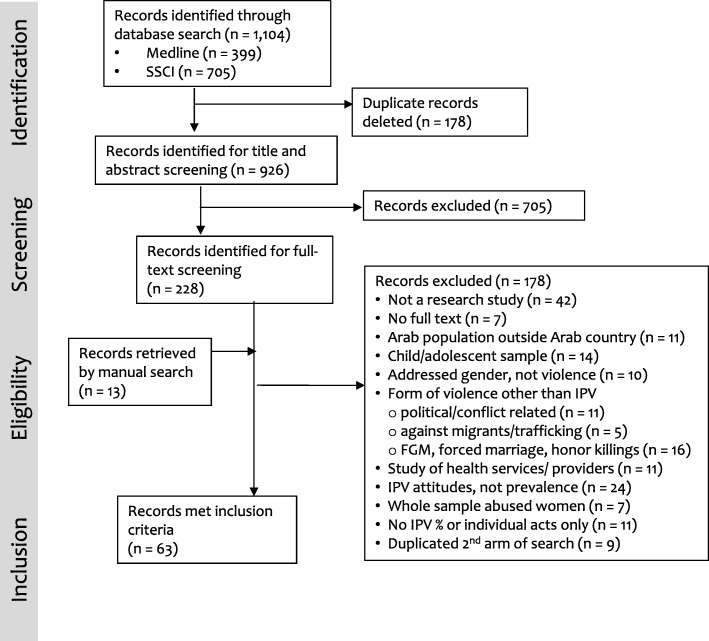
Flowchart: Systematic search for eligible peer reviewed journal articles (records) with prevalence estimates of violence against women by intimate partners from the Arab region, January 2000 – January 2016

The journal database search identified 1104 records (Fig. 1), of which 926 titles and abstracts were screened (after duplicates removed); 228 were selected for full-text screening, along with 13 records retrieved through manual searches. Full text review excluded articles that did not present IPV prevalence data or meet the other inclusion criteria noted earlier. Full text review also excluded nine articles that duplicated DHS datasets retrieved in the second arm of the search – including seven secondary analyses of the 2005 Egypt DHS and two secondary analyses of the 2007 Jordan DHS. Articles reporting on the Palestine 2005/6 national survey (also retrieved in the second search arm) were included, however, because they reported findings more completely than the national report. After full text review was complete, 63 articles that met inclusion criteria remained.

The second search arm identified 11 eligible national, population-based surveys, including five DHS surveys and six governmental surveys. National survey reports from Algeria 2005 [18] and Syria 2005 [19] were not included in the group of 11 eligible national surveys due to inadequate description of methods.

Eleven countries had at least one IPV prevalence survey from either search arm; ten had an eligible study from a journal, and seven had an eligible national, population-based survey published by DHS and/or a government (Table 1). Countries with the highest number of individual surveys (not publications) were Egypt (*n* = 16), Jordan (*n* = 13), Saudi Arabia (*n* = 6), Lebanon (*n* = 4) and Palestine (*n* = 4).

**Table 1:** Number of eligible IPV sources and surveys published 2000–2016, by country and type

Country	1st search arm			2nd search arm	Individual surveys *(any survey or publication type)*
	Peer reviewed journal articles			National survey reports published by DHS or governments	
	Total *(any type)*	*Population-based*	*Facility-based*		
Comoros	0	0	0	1	1
Egypt	18	11	7	3	16
Iraq	3	0	3	1	4
Jordan	19	4	15	2	13
Lebanon	6	1	5	0	4
Morocco	2	0	2	1	3
Palestine	4	4	0	2‡	4
Saudi Arabia	6	0	6	0	6
Sudan	2	1	1	0	2
Syria	3	1	2	0	2
Tunisia	0	0	0	1	1
Total	63	22	41	11	56

‡ The national 2005/6 Palestine survey was retrieved in both arms; rates are included in journal tables

### Characteristics of eligible prevalence sources – risk of bias

Characteristics of eligible surveys from both search arms are presented in Table 2 and Table 3, and described below.

**Table 2:** Characteristics of ***population-based***, household IPV surveys

Country	Lead author, year of publication^a^	Ref.	Year data collected	Dedicated or module	Data collection method	Geographic coverage	Sample size *Women unless noted*	Women’s characteristics *Age, partnership, other*
**From peer-reviewed journals**								
Egypt	Diop Sidibe 2006	[20–22]	1995	Module	FF	National (DHS)	7122	15–49, currently married
	Habib 2011	[23]	2009	Dedicated	FF	Rural area, El-Minia	772	16–50, married
	Hassan 2004	[24–26]	NA	Dedicated	FF	Urban, El-Sheik Zayed, Ismailia	631	15–49, married^b^ with a child < 18 years;
	Seedhom 2012	[27]	2010	Dedicated	FF	Rural area, El-Minia	1502	18–65, ever-married
	Yount 2014	[28, 29]	2012	Module	FF	Rural area, Minya	539	22–65, ever married
	Yount 2005	[30]	1995/7	Module	FF	7 districts, Minya	2522	15–54, ever-married
Jordan	Al-Badayneh 2012	[31]	2006	Dedicated	FF	National	1854	Age not provided, married
	Khawaja 2005	[32, 33]	1999	Module	FF	12 Refugee camps	262 women 133 men	15+, married, Palestinian refugees living with husband
	Shakhatreh 2005	[34]	NA	Dedicated	FF	South Jordan	1007	15–49, any marital status
Lebanon	Khawaja 2004	[35]	1999	Module	FF	Palestinian refugee camps/communities	417 women 417 men	15+, married Palestinian refugees; husbands 20+
Palestine	Haj-Yahia 2000	[36, 37]	1995	Dedicated	SA^c^	National	1334	17–69, married ≥1 year
	Haj-Yahia 2000	[36, 37]	1994	Dedicated	SA^c^	National	2410	17–65, married ≥1 year
	Clark 2010	[38, 39]	2005/6	Dedicated	FF	National	3500	15–64, currently married
Sudan	Ali 2014	[40]	2014	Dedicated	FF	Kassala	1009	15–49, married ≥1 year
Syria	Tappis 2012	[41]	2009	Module	SA	National (refugee communities)	486	‘Reproductive age’, living with husband, Iraqi refugees
**From DHS and government surveys reports**								
Comoros	DGSP 2014	[42]	2012	Module	FF	National	2012	15–49, ever married/cohabited
Egypt	MoHP 2015	[43]	2014	Module	FF	National	6693	15–49, ever married
	El-Zanaty 2005	[44]	2005	Module	FF	National	5613	15–49, ever married
Jordan	DOS 2013	[45]	2012	Module	FF	National	7027	15–49, ever married
	DOS 2008	[46]	2007	Module	FF	National	3444	15–49, ever married
Egypt	Duvvury 2016	[47]	2015	Dedicated	FF	National	18100	18–64, ever married
Iraq	MOH, Iraq 2007	[48]	2006/7	Module	FF	National	14675	15–49, currently married
Morocco	HCP 2012	[49]	2009	Dedicated	FF	National	6712	18–64, currently married
Palestine	PCBS 2012	[50]	2011	Dedicated	FF	National	5811^d^	No age limit, ever married
Tunisia	ONFP 2010	[51]	2010	Dedicated	FF	National	3873	18–64, married/engaged; sexual IPV: married

*Ref* Reference(s), *FF* Face-to-face interviews, *SA* Self-administered questionnaires, *NA* Not available

a. The lead author for only one source is listed per survey; The Ref. # column lists all sources

b. Indicators of past year IPV limited to 590 women living with a husband at the time of the interview

c. Self-administered except for women who were illiterate

d. The number of households was 5811; sample size of eligible women not provided

**Table 3:** Characteristics of ***facility-based*** IPV studies reported in peer-reviewed journal articles

Country	Lead author, publication year^a^	Ref.	Year data collected	Sample design, data collection method	Type of facility, location	Sample size	Women’s characteristics *Age, partnership, other*
Egypt	Bakr 2005	[52]	2004	Convenience; FF	Outpatient clinics, Ain Shams University Hospitals	509	16–65, ever-married
	Elnashar 2007	[53]	2002/3	Systematic; FF	Clinics and hospitals, Dakahlia	936	16–49, currently married
	Fahmy 2008	[54]	2007	Multi-stage, random; FF	Health facilities, Zagazig	500	18–50, currently married
	Guimei 2012	[55]	2007/9	Systematic random; FF	Public health centres, rural and urban Alexandria	450	18+, married with children
	Ibrahim 2015	[56]	2010/12	All women, FF	Obstetric outpatient clinic, Suez Canal University Hospital	1857	18–43, pregnant
	Mamdouh 2012	[57]	2009/10	Random; FF	12 family health centres, rural and urban Alexandria	3271	16–68, ever-married
	Sayed 2014	[58]	2012	Convenience; FF	Primary health care units, Rod-Elfarag and El-Darrasa	187*	Mean age 33^c^, ever-married
Iraq	Ahmed 2012	[59]	2010	Convenience; FF	14 antenatal care units, Erbil City, Kurdistan region	1000	14–48, 6–8 weeks postpartum
	Al-Tawil 2012	[60]	2011	Convenience; SA	3 Christian churches, Ankawa; maternity hospital, Erbil	500	Married ≥1 year^c^; 250 Christian; 250 (mostly Muslim) Kurdish
	Al-Atrushi 2013	[61]	2009/11	Convenience; FF	Reproductive health facilities, Erbil city, Kurdistan region	800	17–65, ever-married, Kurdish
Jordan	Al-Modallal 2010	[62, 63]	NA	Convenience; SA	Higher education institutions	101	25+, married/engaged, working at a university
	Al-Modallal 2012	[64–66]	NA	Convenience; SA	Health centres, refugee camps, Amman, Irbid and Zarqa	300	16–62, ever-married/ engaged, literate Palestinian refugees
	Al-Modallal 2014	[67]	NA	Convenience; SA	Health centres, Amman, Zarqa, Irbid, including refugee camps	620	< 61, married/engaged, not in acute pain or receiving mental health treatment
	Al-Natour 2014	[68]	2011	Stratified random; SA	10 health centres, 3 public hospitals, northern city	80	Mean age 32^b^, married, Jordanian nurses
	Al-Nsour 2009	[69]	2006	Systematic random; FF	Public health centres, Balka	356	18–40, ever-married
	Clark 2009	[70–74]	2005	Systematic; SA	7 reproductive health clinics, north, central, south Jordan	517	15–49, ever-married, literate
	Okour 2011	[75]	2007	Not specified; FF	4 antenatal clinics, Al-Mafraq	303	Mean age 28^b^, pregnant, Bedouin
	Oweis 2010	[76]	2006	All women; SA	5 Maternal and Child Health Centres, Irbid City	316	15–45, pregnant
Lebanon	Awwad 2014	[77]	NA	Convenience; FF	Gynaecological services, American University of Beirut Medical Centre	91	20–65, ever married
	Hammoury 2007	[78–80]	2005	Convenience; FF	Primary health care clinics, Sidon	349	15–42, married, pregnant, Palestinian refugees
	Usta 2007	[81]	2002	Convenience; FF	4 primary health care centres	1415	14–65, any marital status
Morocco	Manoudi 2013	[82]	2006	Not specified; SA^b^	Health centre, Marrakech	265	18–65, married
	Boufettal 2012	[83]	2008/9	Not specified, FF	University hospital, Casablanca	867	14–44, any marital status, pregnant
Saudi Arabia	Afifi 2011	[84]	2010	Cluster random; FF	Primary health care centres, Al-Ahsa, urban and villages	2000	16–59, ever-married
	Al-Faris 2013	[85]	2009/10	Convenience; SA^b^	Outpatient clinics, King Khalid University Hospital, Riyadh	222	15–70, married
	Eldoseri 2014	[86]	2012	Convenience; FF	Primary-care centres, Jeddah	200*	18–65, ever-married
	Fageeh 2014	[87]	2011/12	Convenience; SA^b^	3 tertiary hospitals, Jeddah	2301	15–70, ever-married
	Rachana 2002	[88]	1996/99	All women^d^	2 university teaching hospitals	7105	Pregnant, leading to live, singleton birth^c^
	Tashkandi 2009	[89]	2004	Simple random; FF	16 primary health centres, Medina	689	16–60, ever-married, not widowed, Saudi Arabian
Sudan	Ahmed 2005	[90]	2001/2	All women; SA	Arda Medical Centre, Omdurman	394	Married, literate^c^
Syria	Maziak 2002	[91, 92]	NA	Random, FF	8 primary care centres, Aleppo	412	13–61, any marital status

*Ref* Reference(s), *FF* Face-to-face interviews, *SA* Self-administered questionnaires

*Sample size < 300 but justified with a published calculation of precision

a. The lead author for only one source is listed per survey; The Ref. column lists all sources

b. Administered with assistance (face-to-face) if needed, e.g. in cases of illiteracy

c. Age range unspecified

d. Violence was measured by self-report, but article did not specify whether this meant face-to-face or self-administered

#### Representativeness: study and sample design

Population-based household surveys – considered the gold standard for IPV prevalence research [16] – comprised almost one-third (*n* = 15) of the 46 individual IPV surveys reported in journals; two-thirds (*n* = 31) were facility-based. All 11 national surveys from the second search arm were population-based by definition. Distribution by type of survey varied by country. Saudi Arabia had six eligible facility-based studies but no population-based studies. Palestine had four population-based but no facility-based studies. All population-based surveys used multi-stage, cluster sampling, though some older surveys reported an inadequate sampling frame [93]. A majority of facility-based studies used convenience samples, but some used systematic or random designs or interviewed all women attending health services during a given period.

Except for five facility-based and one population-based study, most surveys met the sample size quality criteria of ≥ 300 or provided reasonable justification for the smaller sample size [58, 86]. Samples sizes varied widely, from 80 to 7105 in journals and from 2012 to 18100 in national DHS and governmental surveys. Almost all surveys limited IPV samples to ever- or currently-partnered women, except for studies from Jordan [34] and Syria [91] that included all women, regardless of partnership. Some samples were limited further, e.g. to women living with a husband or child < 18, refugees or pregnant women.

#### Operational definitions of partners, perpetrators and forms of violence

Reflecting conservative social norms in the Arab region, almost all surveys defined a partner as a husband except for a few that included fiancés, the Comoros DHS [42], which included any cohabiting partner, and a study from Morocco that did not specify [83]. Surveys fell into four categories: those that asked about violence by the current partner; by the current/most recent partner; by any partner in life; and by any household member. Recent DHS surveys from Comoros, Egypt, and Jordan [42, 43, 45] measured violence both by the current/most recent partner and by any partner in life. Several studies [20, 41, 78, 81, 84, 87, 91] used measures that were not partner specific, i.e. they asked about violence by any family member or anyone, and relied on follow-up questions to determine whether the perpetrator was a partner. Previous work [15] suggests this approach may underestimate prevalence.

The majority (*n* = 32) of the 46 surveys reported in journals were published with operational definitions of each form of violence measured (Additional file 1). Articles reporting on seven surveys did not provide operational definitions but did name or describe instruments used; this information provided a general idea of how violence was defined, but could not account for changes that may have been made during adaptation or translation.

Among the 21 surveys reported in journals that used or adapted international instruments, at least ten used WHO instruments [23, 27, 60, 61, 71, 75, 84–86], three used DHS questionnaires [20, 28, 30], and four [66–68, 78] used the Abuse Assessment Screen (AAS) or the Women’s Abuse Screening Tool (WAST) (Additional file 1). DHS reports included definitions and full questionnaires. Some (e.g. Tunisia [51]), but not all national government survey reports provided operational definitions, and no governmental report included a questionnaire – at least not in versions published online.

#### Measures of physical violence

Most surveys (*n* = 39) from journals and all national surveys measured physical IPV. Most used measures that aligned with international guidelines [16] by asking women about a behaviourally specific list of acts (e.g. slapped, kicked, beat up, etc.). However, several studies [20, 30, 55, 82] limited measures of physical IPV to “beaten”. Surveys that used the AAS or WAST asked women about abstract concepts such as ‘violence’ or ‘abuse’ – an approach known to underestimate IPV prevalence [15, 94]. One study from Jordan [69] asked an open-ended question about the most serious form of violence experienced in the past year. Two surveys, one from Egypt [58] and one from Morocco [83] included forced sex in rates of physical violence. One study from Jordan [31] may have included witnessing, not just experiencing violence in prevalence rates. A study from Syria [91] required a threshold of ≥ 3 experiences in the past year and ≥ 1 in the past 30 days to classify women as having experienced IPV.

#### Measures of sexual violence

Just over half (*n* = 25) individual studies from journals measured sexual violence. Some researchers said the topic was too sensitive; researchers from Sudan removed questions about sexual violence after the first 15 respondents refused to give clear answers [90]. Most surveys defined sexual IPV as forced sexual intercourse and/or sex acts. Surveys using the WHO instrument also measured unwanted sex due to fear of a their partner might do if they refused. A few surveys measured less common acts, such as being denied sex or hurt during sex [23], being prevented from using contraception [38] or being told hurtful things during sex [57]. Studies using AAS or WAST asked about sexual ‘abuse’ rather than behaviourally specific acts.

#### Measures of emotional/psychological abuse

Two-thirds of surveys from journals measured emotional/psychological IPV, but definitions varied widely. Many studies – including most using DHS or WHO instruments – defined ‘emotional’ IPV to include insults, humiliation, intimidation and threats to harm her or someone close to her. Other commonly measured acts included threats to divorce, to take a second wife, to expel her from the home or to take away her children. While most studies classified threats with a weapon as physical violence, others, including from Egypt [52, 58] and Saudi Arabia [89] classified such acts as emotional/psychological or “threat” violence.

Some surveys measured less common acts of emotional/psychological abuse. The WorldSAFE study from Egypt [26] asked women whether their husband had ever abandoned them without financial support for at least 6 months and whether he had ever been unfaithful. Another study from Egypt [58] asked whether their husbands had “spied” on them. One study from Jordan [62] explored “work-related harassment” by partners, such as harassing phone calls at work. Studies using the AAS asked women whether they were “afraid of someone in the household” and classified that as emotional abuse. The national survey from Tunisia [51] measured two acts that straddled emotional and sexual abuse: no longer wanting to have sex with her; and bringing other women (mistresses) to the home. Two studies from Egypt [26, 91] limited emotional/psychological abuse with frequency thresholds, e.g. ≥ 3 times in a specific time period.

There was little consistency across surveys about terms used to classify acts as ‘emotional’, ‘psychological’, ‘threat’, ‘verbal’, or ‘economic’ abuse, or ‘control’ (Additional file 1). Some [77, 81] classified insults, threats, or degrading remarks as ‘psychological’ or ‘verbal’ abuse, separate from ‘emotional abuse’; others had a distinct category of ‘threat’ violence. Some studies classified controlling behaviours (e.g. limiting women’s movements or contacts with family and friends) as a component of ‘emotional’, ‘psychological’ or ‘social’ violence; while others classified them as a form of IPV labelled ‘control’ that also included insults, humiliation or shouting [52, 58, 90]. DHS surveys considered “controlling behaviours” a related, but separate domain from violence.

Eight studies from journals measured acts related to money, such as preventing women from having or earning money, taking their earnings, or denying them financial support. Three surveys [52, 54, 58] classified such acts as a component of ‘control’ or ‘social’ abuse; five [36, 57, 66, 77, 81] classified them as a distinct category of ‘economic’ abuse.

#### Prevalence indicator construction

Most surveys constructed prevalence indicators in accordance with international good practice guidelines [16]; however, some articles did not clarify whether timeframe was lifetime, past year, or other [34, 66, 67]. Some reported rates of violence by any household/family member, not just partners [41, 81, 84, 87, 88, 92]. Habib et al. [23] reported the percentage of women who disclosed emotional abuse only, but no physical or sexual abuse. Similarly, Tashkandi and Rasheed [89] reported the prevalence of emotional abuse only with no physical IPV. Given that abused women often experience multiple forms of IPV, these indicators captured only a fraction of those who reported ***any*** physical or ***any*** emotional IPV. Al-Nsour and colleagues [69] reported the percentage of women who reported that the most serious violence in the past year was physical, rather than the prevalence of ***any*** physical IPV (same for emotional IPV).

#### Safety and ethics

While most sources described informed consent or safety procedures, only about one-fourth (12 of 46) of studies reported in journals mentioned adherence to WHO Ethical and Safety Recommendations for Research on Domestic Violence Against Women [17]. Studies from Lebanon [35] and Egypt [20] did not adhere to WHO guidelines by: a) interviewing matched members of couples about IPV; b) interviewing more than one woman in the home; and/or c) allowing children to be present during the interview. Among the 11 reports presenting findings of national surveys, four affirmed adherence to WHO guidelines; four did not but did describe field procedures that met those recommendations; two did not provide enough information to assess; and one from Morocco [49] described field procedures that did not correspond with WHO guidelines (e.g. more than one in five women may have been interviewed in front of a third person).

### IPV prevalence reported in journals

#### Physical IPV prevalence

Rates of physical IPV (ever) reported in journals ranged from 6 to 43% in population-based studies (Table 4), and from 12 to 59% in facility-based studies (Table 5). About half of the studies documented a prevalence of nearly one-third or more. Past-year rates of physical IPV ranged from about 5% to over 50% in population-based studies and from 15 to 26% in facility-based studies. Neither of the two articles reporting the lowest rates of physical IPV (5–6%) [34, 41] published detailed operational definitions or clearly explained their indicator construction.

**Table 4:** Percentage of women reporting IPV, ever and past year, by type, ***population-based*** surveys from journals

Country	Lead author, publication year	Year(s) data collected	Risk of bias¥ score	Physical		Sexual		Emotional/psychological		Physical, sexual and/or emotional/ psychological *unless noted*	
				Ever	Past year	Ever	Past year	Ever	Past year	Ever	Past year
				%	%	%	%	%	%	%	%
Egypt	Diop Sidibe 2006	1995	3	34	16^a^	–	–	–	–	–	–
	Habib 2011	2009	3	29.9^b^	–	7.8^c^	–	6.6^d^	–	57.4	–
	Hassan 2004	NA	2	11.2	10.5	–	–	10.5 severe	10.8^e^ severe	–	–
	Seedhom 2012	2010	1	30.3	–	7.3	–	49.3	–	60.4	–
	Yount 2014	2012	3	–	–	–	–	–	–	67^f^	–
	Yount 2005	1995/97	4	26.8	9.1	–	–	–	–	–	–
Jordan	Al-Badayneh 2012	2006	4	–	–	–	–	–	–	–	98
	Khawaja 2005	1999	3	42.5^g^	19.2	–	–	–	–	–	–
	Shakhatreh 2005	NA	6	5 (timeframe unspecified)		–	–	10 (timeframe unspecified)		–	–
Lebanon	Khawaja 2004	1999	5	22.0^h^	9.1^i^	–	–	–	–	–	–
Palestine	Haj-Yahia 2000	1994	3	–	52	–	37.6	–	91	–	–
	Haj-Yahia 2000	1995	3	–	54	–	40	–	87.2*	–	–
	Clark 2010	2005/6	2	–	22.2	–	10.6	–	61.6	–	–
Sudan	Ali 2014	2014	3	–	33.5	–	17.0	–	30.1 psych. 47.6% verbal	–	–
Syria	Tappis 2012	2009	5	6*‡	4.7*‡	–	–	14.6*‡ verbal 5.1*‡ emotional	11.5*‡ verbal 3*‡ emotional	30P/E‡	17P/E‡

¥Risk of bias was scored from 0 to 11 with 0 meaning the least risk of bias and 11 meaning the highest

*Percentage calculated by authors based on published numbers of women reporting that form of IPV

‡Included violence by any household/family members, including husbands

*P/E* Physical and/or emotional

a. Figure for currently married women extracted from Diop Sidibe et al. [20]

b. Percentage of women who reported physical violence only with no sexual or emotional violence

c. Sexual only, no emotional or physical

d. Emotional only, no physical or sexual

e. Limited to women currently living with a husband

f. Rate for physical and/or sexual violence ever was 54%

g. Husbands reported 48.9%

h. 29.5% of husbands reported physical violence against wives; in 32.9% of couples, at least one member reported physical IPV against wives

i. 10.4% of husbands reported sexual IPV against wives; in 12.9% of couples, at least one member reported sexual IPV against wives

**Table 5:** Percentage of women reporting IPV, ever and past year, by type, ***facility-based*** surveys from journals

Country	Lead author, publication year	Year(s) data collected	Risk of bias¥ score	Physical		Sexual		Emotional/psychological		Physical, sexual or emotional/ psychological *unless noted*	
				Ever	Past year	Ever	Past year	Ever	Past Year	Ever	Past year
				%	%	%	%	%	%	%	%
Egypt	Bakr 2005	2004	6	56	–	17.1	–	47.9 threats 88.4 control	–	89.8	–
	Elnashar 2007	2002/3	5	–	–	–	11.5	–	–	–	–
	Fahmy 2008	2007	4	22.4	–	19.6	–	74 psych. 26.8 social	–	62.2	–
	Guimei 2012	2007/9	5	53.6*	–	31.8*	–	–	–	–	–
	Mamdouh 2012	2009/10	6	50.2	–	37.1	–	71.0 emotional 40.8 economic	–	77	–
	Sayed 2014	2012	7	57	–	9.6	–	50.3* threats 55.6* control	–	75.9	–
Iraq	Ahmed 2012	2010	7	–	–	–	–	–	–	11.8* P/S	–
	Al-Tawil 2012	2011	4	–	17.6	–	9.4	–	32.4	–	–
	Al-Atrushi 2013	2009/11	3	38.9	15.1	21.1	12.1	52.6	43.3	58.6	45.3
Jordan	Al-Modallal 2010	NA	7	–	–	–	–	48.5 work-related	–	–	–
	Al-Modallal 2012	NA	6	22.7^a^	–	16.7^a^	–	50.3^a^ emotional 73.7^a^ control 53.3^a^ economic		78^a^ 43.4^a^ P/S	
	Al-Modallal 2014	NA	8	–	–	–	–	–	–	35.3*^a^	
	Al-Natour 2014	2011	7	12.5	–	5.1	–	59.0	–	–	–
	Al-Nsour 2009	2006	5	–	19.6†	–	–	–	47.5† emotional 12.3† neglect	–	87† P/E
	Clark 2008	2005	2	31.2^c^	–	18.8^c^	–	73.4^c^ psych. 97.2^c^ control	–	38^c^ P/S	–
Lebanon	Awwad 2014	–	5	40.6	–	33.0	–	64.8 verbal 19.0 emotional 22.0 social	–	–	–
	Hammoury 2007	2005	5	59‡	19.1‡	–	26.2	–	16‡	68.8‡	–
	Usta 2007	2002	5	23‡	–	–	–	31‡ insults^d^	–	35‡ P/E	–
Morocco	Manoudi 2013	2006	8	16.6	–	–	–	–	–	–	–
Saudi Arabia	Afifi 2011	2010	5	12.1*^e^	–	5.6*^e^	–	19.8*^e^	–	–	–
	Al-Faris 2013	2009/10	6	12.2	–	–	–	–	–	–	–
	Eldoseri 2014	2012	4	44.5	16.0	–	–	–	–	–	–
	Fageeh 2014	2011/12	5	11.6‡	–	4.8‡	–	29‡	–	34‡	–
	Tashkandi 2009	2004	6	26.9	25.7	–	–	57.8 shouting^f^	57.5*^g^	57.8^h^	58.5
Sudan	Ahmed 2005	2001/2	5	–	20.1*	–	–	–	28.4* control 30.2* threats of harm	–	41.6
Syria	Maziak 2002	–	4	–	23.1^i^‡	–	–	–	–	–	–

¥Risk of bias was scored from 0 to 11, with 0 meaning the least risk of bias and 11 meaning the highest

*Percentage calculated by authors based on published numbers of women reporting that form of IPV

*P/S* Physical and/or sexual, *P/E * Physical and/or emotional

†Based on most serious act of “abuse” in past year, measured with an open-ended question

‡Included violence by any household/family members, including husbands

a. Timeframe unclear

b. 52.7% of women ***not*** receiving mental health treatment reported emotional IPV

c. Articles reported different rates depending on subsamples of interest. Figures in this table come from Clark et al. [71]

d. Also, threats of harm (15%); divorce (4%); to take away children (12%); denied financial support (12%)

e. Violence by any family member (ever) was 17.9% physical; 6.9% sexual; and 35.9% emotional

f. Article did not report rates of ***any*** emotional violence ever; 30.9% reported emotional violence with no physical violence

g. 32.8% women reported experiencing emotional but no physical IPV in the past year

h. Physical and/or emotional IPV. Article did not explain how lifetime prevalence could be lower than past year

i. 26% among married women

#### Sexual IPV prevalence

Rates of lifetime sexual IPV reported in facility-based studies ranged from 5 to 37%. Only two population-based studies from journals measured lifetime sexual IPV, reporting rates between 7 and 8%. Past year sexual IPV ranged from 11 to 40% in population-based studies and 9 to 26% in facility-based studies.

#### Emotional/psychological IPV prevalence

Lifetime rates of emotional/ psychological abuse (however defined) were 19 – 74% in facility-based studies and 5 – 49% population-based studies; past year rates ranged from 16 to 58% in facility-based studies and 3 to 91% in population-based studies.

#### Prevalence of economic abuse

In five studies that measured economic abuse as a separate form of IPV, rates ranged from 12% in a study from Lebanon [81] that defined economic abuse as ‘denied financial support’ to 53% in a study from Jordan [66] that defined economic abuse as preventing her from knowing about/accessing family income and/or preventing her from working outside the home.

### IPV prevalence from national DHS and governmental surveys

Excluding Comoros, national estimates of physical IPV ranged from 20% in Tunisia to 33% in Egypt, ever; and from 6% in Morocco to 24% in Palestine, past year (Table 6). The 2012 Comoros DHS [42] produced the lowest national estimates of physical IPV, both ever and past year – 7.3 and 4.3% respectively. National estimates of sexual IPV ever ranged from 2% in Comoros to 14% in Tunisia; while past year estimates ranged from 1% in Comoros to 12% in Palestine. Estimates of emotional/psychological IPV ranged from 8.1% in Comoros to 33.4% in Iraq, ever, while past year estimates ranged from 6.2% in Comoros to more than half in Palestine.

**Table 6:** Percentage of ever-partnered^*^ women reporting IPV ever and past year, ***population-based*** DHS or governmental surveys

Country	Year data collected	Risk of bias‡	By which partner?	Physical		Sexual		Emotional		Physical and/or sexual	
				Ever	Past year	Ever	Past year	Ever	Past year	Ever	Past year
				%	%	%	%	%	%	%	%
**DHS Surveys**											
Comoros	2012	2	Any in life	7.3	4.3	3.3	1.3	–	–	8.4	4.9
			Current/most recent	5.6	4.2	1.8	1.3	8.1	6.2	6.4	4.9
Egypt	2014	0	Any in life	25.7	13.5	4.5	2.7	–	–	26.0	14.0
			Current/most recent	25.2	13.5	4.1	2.7	18.8	13.1	25.6	14.0
	2005	1	Current/most recent	33.2	20.4	6.6	3.8	17.5	10.7	33.7	20.4
Jordan	2012	1	Any in life	21.8	11.2	9.2	6.0	–	–	24.3	14.1
			Current/most recent	21.1	11.2	8.6	6.0	24.6	17.4	23.6	14.1
	2007	1	Current/most recent	20.6	12.2	7.6	5.6	20.0	14.0	23.0	14.6
**National, governmental surveys**											
Egypt	2015	0	Current/most recent	31.8	11.8	12.3	6.5	42.5	22.3	34.1	14.0
Iraq	2006/7	1	Current	–	21.2	–	–	–	33.4	–	–
Morocco	2009	3	Current	–	6.4	–	6.6	–	38.7	–	11.5
Palestine	2011	3	Current	–	23.5	–	11.8	–	58.6	–	–
Tunisia	2010	0	Current	20.3	7.2	14.2	9.0	24.8	17.0	–	–

*Surveys defined ‘partner’ as a husband, except 2012 Comoros DHS, which included cohabiting unmarried partners

‡Risk of bias was scored from 0 to 11, with 0 meaning the least risk of bias and 11 meaning the highest

### IPV during pregnancy

In seven facility-based studies, currently pregnant women reported rates of physical violence during pregnancy ranging from 10.4 to 34.6% (Table 7). DHS surveys from Comoros [42], Egypt [43] and Jordan [45] measured physical violence by any perpetrator during any pregnancy in life among ever pregnant women, and found levels of 3, 7 and 7%, respectively.

**Table 7:** Percentage of currently pregnant women reporting physical, sexual or emotional/psychological ***IPV during pregnancy***

Country	Lead author, publication year	Year data collected	Risk of bias‡ *score*	Violence during pregnancy				Perpetrator, timeframe
				Physical	Sexual	Emotional/ psychological	Physical, sexual and/or emotional/ psychological	
				%	%	%	%	
Egypt	Ibrahim 2015	2010/12	2	15.9	10.0	32.6	44.1	Husband, current pregnancy
Jordan	Clark, 2009	2005	1	15.4	–	–	–	Anyone, any pregnancy
	Okour 2011	2007	3	34.6	15.5	28.1	40.9	Husband, current pregnancy
	Oweis 2010	2006	4	10.4	5.7	23.4 emotional 23.7 verbal	–	Husband, current pregnancy
Lebanon	Hammoury 2007	2005	4	11.4	–	–	–	Husband/other household member, current pregnancy
Morroco	Boufettal 2012	2008/9	4	12.3 physical/sexual		–	–	Intimate partner
Saudi Arabia	Rachana 2002	1996/99	5	20.9	–	–	–	Husband or in laws, current pregnancy

‡Risk of bias was scored from 0 to 11, with 0 meaning the least risk of bias and 11 meaning the highest

## Discussion

This systematic review found uneven geographic coverage and quality of evidence on IPV against women in the Arab region. Only half of the 22 Arab countries had eligible prevalence surveys, disproportionately from Egypt and Jordan. A majority of surveys were facility-based, known to have limited generalizability to the broader population of women [16]. Some governments (e.g. the State of Palestine) had invested in repeated, population-based VAW national data collection, but relatively few Arab countries had even one round of national IPV prevalence estimates, and only three countries (Comoros, Egypt and Jordan) — those with DHS violence modules — had estimates included in the SDG global database.

Evidence, however fragmented, suggests that IPV against women in Arab countries represents an important public health and human rights problem. Reported IPV rates varied widely across surveys, but generally aligned with WHO estimates of 37% of physical and/or sexual IPV against ever-partnered women in (at least a portion of) the region [7]. In DHS surveys, 3 – 7% of ever pregnant women reported violence during any pregnancy in life by any perpetrator, which fell within the 2 – 14% range documented by a 2010 global review [95]; but facility-based studies from the region documented substantially higher rates of IPV during pregnancy among ***currently*** pregnant women, ranging from 10 to 35% — possibly due to better recall or selection bias among women receiving prenatal care compared with the general population.

Wide variations in reported prevalence likely reflects the diversity of methods and operational definitions across surveys, particularly from the journal literature, suggesting a need for caution when comparing prevalence rates across surveys, especially when measures do not conform to international guidelines [16] or were not operationally defined. Cross-national comparisons may not be feasible until countries develop more comparable prevalence estimates. Definitions of emotional/psychological IPV were the most varied, rendering comparisons across studies impossible without careful examination of acts measured. This diversity persisted even across studies that adapted the WHO instrument. For example, Clark and colleagues [71] found that prevalence rates analysed with their study-specific definitions were substantially higher than rates produced with WHO definitions: 73% vs. 50% (respectively) for lifetime psychological violence and 97% vs. 83% (respectively) for controlling behaviours. Diverse definitions of emotional/psychological IPV are not unique to Arab countries, but have been noted by global reviews [7, 96].

### Gaps in the regional evidence about IPV

This review highlights gaps in the regional evidence base. One gap is a lack of data on sexual IPV. While all national surveys measured sexual IPV prevalence, only about half the surveys from journals did so, often describing the topic as too sensitive. Given that the Egypt DHS has measured sexual IPV since 2005, it should be feasible to expand data collection in this area, perhaps by encouraging researchers to share lessons learned within the region.

SDG Indicator 5.2.1 metadata [10] notes that the most important sources of national IPV prevalence data are “specialized national surveys dedicated to measuring violence against women and international household surveys that include a module on experiences of violence by women,” such as DHS. Most journal articles with IPV data reported findings of subnational surveys, which cannot monitor national levels of violence.

Generally there is a need for more comparable, high quality, nationally-representative IPV data to allow Arab countries to monitor levels of IPV over time and report on SDG Indicator 5.2.1. Such data would also allow researchers to examine cross-national associations between IPV prevalence and factors related to gender equality, including legal frameworks and levels of child marriage. Barriers to national data collection may include political instability, armed conflict or forced displacement, lack of national commitment to address VAW, and/or the challenges of carrying out national, household survey data collection on sensitive topics.

DHS violence modules and the WHO VAW instrument offer Arab countries a way to expand the evidence base in a comparable way. While the search did not identify any official WHO surveys in Arab countries, WHO instruments were mentioned by nearly one-fourth of surveys from journals and several national surveys. WHO is currently revising the instrument; ideally the next iteration will be translated into Arabic, adapted to the cultural context and shared in the region.

### Limitations

Prevalence estimates summarized in this review may underestimate ‘true’ IPV prevalence due to barriers to disclosure. Widespread social norms in the Arab region support husbands’ right to physically discipline wives, and abused women often face high social, economic and legal barriers to divorce and unresponsive law enforcement and health care institutions [4]. Women are often reluctant to report violence to authorities [97] and may hesitate to disclose violence to survey interviewers.

This review had other important limitations. First, it did not include a comprehensive search of the grey literature and may have missed valuable subnational studies not published in journals. Second, while basic reporting quality (clear description of research design, study population, sample size and respondent selection) was an eligibility criterion, many studies had other risks of bias. This provided a more comprehensive view of the regional evidence, but it means that these prevalence rates should be interpreted with caution. In particular, facility-based surveys – especially those with convenience samples – are not necessarily generalizable to the broader population. Additionally, field procedures, interviewer selection and training may have affected data quality and women’s willingness to disclose violence [98], but these are difficult to assess from published reports alone.

Another limitation is that this review did not include a meta-analysis or a quantitative analysis of how methodological characteristics correlated with differences in rates across studies. Given the complexity of the data and lack of consensus about definitions, such analyses were not feasible given the scope of this paper, but are worthy of future attention. Finally, this review did not examine attitudes, help-seeking behaviours, consequences, risk/protective factors, or social determinants of partner violence – all important topics for future research.

## Conclusions

IPV against women is a global phenomenon that thrives in a culture of silence. IPV has particular importance in the Arab region, given norms and systems that reinforce male authority over women [4]. Though data are fragmented and often not comparable across sources, evidence suggests that the problem is substantial, and there is a need for greater investment in violence prevention and response. There is also need for more high quality data collection and analysis in Arab countries, to understand the magnitude of IPV, monitor change over time, and explore pathways that perpetuate violence and prevent an adequate response. Researchers also need greater familiarity with international standards for ethical research on IPV. Countries need more nationally-representative IPV estimates, such as dedicated violence surveys modelled on the WHO multi-country study or from violence modules embedded in recurring surveys such as the DHS. A stronger evidence base could inform more effective policies and programmes by breaking the silence, raising awareness, engaging men and women to mobilize action and helping countries monitor progress towards the SDG vision of healthier, more peaceful families and societies.

## Supplementary information

Additional file 1: Operational definitions of physical, sexual, and/or emotional IPV in peer reviewed articles from the journal literature from Arab countries, 2000–2016. (DOCX 30 kb)

## Abbreviations

AAS: Abuse Assessment Screen
DHS: Demographic and Health Survey
IPV: Intimate partner violence
SDGs: Sustainable Development Goals
UN: United Nations
VAW: Violence against Women
WAST: Woman Abuse Screening Tool
WHO: World Health Organization

## Footnotes

1 Algeria, Bahrain, Comoros, Djibouti, Egypt, Iraq, Jordan, Kuwait, Lebanon, Libya, Mauritania, Morocco, Oman, Palestine, Qatar, Saudi Arabia, Somalia, Sudan, Syria, Tunisia, the United Arab Emirates and Yemen.

## References

[1] League of Arab States. http://www.leagueofarabstates.net/ar/aboutlas/Pages/CountryData.aspx. Accessed 16 July 2019.

[2] ILO (2012). Rethinking economic growth: Towards productive and inclusive Arab societies.

[3] World Economic Forum (2017). The global gender gap report 2017.

[4] Women UN (2017). Violence against Women: what is at stake? Status of Arab Women report 2017.

[5] Obermeyer CM, Bott S, Sassine AJ (2015). Arab adolescents: health, gender, and social context. J Adolesc Health.

[6] El Feki S, Heilman B (2017). Barker G (eds.): understanding masculinities: results from the international men and gender equality survey (IMAGES) – Middle East and North Africa.

[7] WHO, LSHTM (2013). MRC: global and regional estimates of violence against women: prevalence and health effects of intimate partner violence and non-partner sexual violence.

[8] UN (2015). Transforming our world: the 2030 agenda for sustainable development. Resolution adopted by the United Nations general assembly on 25 September 2015.

[9] United Nations. Sustainable development goal indicators global database. New York: United Nations. n.d.

[10] UN: SDG Indicators Metadata Repository. New York: United Nations Statistics Division; 2018.

[11] UN Women. Global database on violence against women. New York: UN Women. n.d.

[12] ONA: Open Data Kit (ODK) ONA; n.d.

[13] Munn Z, Moola S, Riitano D, Lisy K (2014). The development of a critical appraisal tool for use in systematic reviews addressing questions of prevalence. Int J Health Policy Manag.

[14] Hoy D, Brooks P, Woolf A, Blyth F, March L, Bain C, Baker P, Smith E, Buchbinder R (2012). Assessing risk of bias in prevalence studies: modification of an existing tool and evidence of interrater agreement. J Clin Epidemiol.

[15] Ellsberg M, Heise L (2005). Researching violence against women: a practical guide for researchers and activists.

[16] UN (2014). Guidelines for producing statistics on violence against women.

[17] WHO (2001). Putting women first: Ethical and safety recommendations for research on domestic violence against women.

[18] INSP (2005). Violences à l’Encontre des Femmes, Enquête nationale.

[19] UNIFEM: Violence against Women study, Syria 2005. UNIFEM and the Syrian Commission for Family Affairs; n.d.

[20] Diop-Sidibe N, Campbell JC, Becker S (2006). Domestic violence against women in Egypt--wife beating and health outcomes. Soc Sci Med.

[21] Akmatov MK, Mikolajczyk RT, Labeeb S, Dhaher E, Khan MM (2008). Factors associated with wife beating in Egypt: analysis of two surveys (1995 and 2005). BMC Womens Health.

[22] Ambrosetti E, Abu Amara N, Condon S (2013). Gender-based violence in Egypt: analyzing impacts of political reforms, social, and demographic change. Violence Against Women.

[23] Habib SR, Abdel Azim EK, Fawzy IA, Kamal NN, El Sherbini AM (2011). Prevalence and effects of violence against women in a rural community in Minia governorate, Egypt. J Forensic Sci.

[24] Jeyaseelan L, Sadowski LS, Kumar S, Hassan F, Ramiro L, Vizcarra B (2004). World studies of abuse in the family environment--risk factors for physical intimate partner violence. Inj Control Saf Promot.

[25] Hassan F, Sadowski LS, Bangdiwala SI, Vizcarra B, Ramiro L, De Paula CS, Bordin IA, Mitra MK (2004). Physical intimate partner violence in Chile, Egypt, India and the Philippines. Inj Control Saf Promot.

[26] Ramiro LS, Hassan F, Peedicayil A (2004). Risk markers of severe psychological violence against women: a WorldSAFE multi-country study. Inj Control Saf Promot.

[27] Seedhom AE (2012). Sociodemographic associations of intimate partner violence against women in a rural area, El-Minia governorate, Egypt, 2010. J Public Health (Germany).

[28] Yount KM, Zureick-Brown S, Salem R (2014). intimate partner violence and women's economic and non-economic activities in Minya, Egypt. Demography.

[29] Yount KM, Dijkerman S, Zureick-Brown S, VanderEnde KE (2014). Women's empowerment and generalized anxiety in Minya, Egypt. Soc Sci Med.

[30] Yount KM (2005). Resources, family organization, and domestic violence against married women in Minya**,** Egypt. J Marriage and Family.

[31] Al-Badayneh DM (2012). Violence against Women in Jordan. J Fam Violence.

[32] Khawaja M, Barazi R (2005). Prevalence of wife beating in Jordanian refugee camps: reports by men and women. J Epidemiol Community Health.

[33] Khawaja M, Linos N, El-Roueiheb Z (2008). Attitudes of men and women towards wife beating: findings from Palestinian refugee camps in Jordan. J Fam Violence.

[34] Shakhatreh FM, Maaitah RM, Gharaibeh MK (2005). Psychosocial functioning and determinants of domestic violence among women. An epidemiological study. Saudi Med J.

[35] Khawaja M, Tewtel-Salem M (2004). Agreement between husband and wife reports of domestic violence: evidence from poor refugee communities in Lebanon. Int J Epidemiol.

[36] Haj-Yahia MM (2000). The incidence of wife abuse and battering and some sociodemographic correlates as revealed by two national surveys in Palestinian society. J Fam Violence.

[37] Haj-Yahia MM (2000). Implications of wife abuse and battering for self-esteem, depression, and anxiety as revealed by the second Palestinian national survey on violence against women. J Fam Issues.

[38] Haj-Yahia MM, Clark CJ (2013). Intimate partner violence in the occupied Palestinian territory: prevalence and risk factors. J Fam Violence.

[39] Clark CJ, Everson-Rose SA, Suglia SF, Btoush R, Alonso A, Haj-Yahia MM (2010). Association between exposure to political violence and intimate-partner violence in the occupied Palestinian territory: a cross-sectional study. Lancet.

[40] Ali AA, Yassin K, Omer R (2014). Domestic violence against women in eastern Sudan. BMC Public Health.

[41] Tappis H, Biermann E, Glass N, Tileva M, Doocy S (2012). Domestic violence among Iraqi refugees in Syria. Health Care Women Int.

[42] DGSP, ICF International (2014). Enquête Démographique et de Santé et à Indicateurs Multiples aux Comores, 2012.

[43] MoHP, El-Zanaty and Associates, ICF International. Egypt Demographic and Health Survey 2014. Cairo: Ministry of Health and Population (MoHP) and ICF International. p. 2015.

[44] El-Zanaty F, Way A (2006). Egypt demographic and health survey 2005 Cairo: Egypt: Ministry of Health and population.

[45] DOS, ICF International (2013). Jordan Population and Family Health Survey 2012.

[46] DOS, Macro International Inc. Jordan Population and Family Health Survey 2007. Calverton: Jordan Department of Statistics (DOS) and Macro International Inc. p. 2008.

[47] Duvvury N, Marcos MO, Gadallah M, Attia S, El Adly N, Maged W, Haddad G (2016). The Egypt Economic Cost of Gender-Based Violence Survey (ECGBVS) 2015.

[48] Iraq MH (2007). Iraq Family Health Survey Report, 2006/7.

[49] HCP (2012). Enquête nationale sur la prévalence de la violence à l’égard des femmes au Maroc [National survey on the prevalence of violence against women] (ENPVEF) 2009.

[50] PCBS (2012). Violence Survey in the Palestinian Society, 2011: Main findings.

[51] ONFP, AECID (2010). Rapport Enquête Nationale Sur la violence à l’égard des femmes en Tunisie [Report on the National Survey on Violence against Women in Tunisia].

[52] Bakr IM, Ismail NA (2005). Domestic violence among women attending out-patient clinics in Ain Shams University hospitals, Cairo, Egypt. J Egypt Public Health Assoc.

[53] Elnashar AM, Ibrahim MED, Eldesoky MM, Aly OM, Mohamed Hassan MES (2007). Sexual abuse experienced by married Egyptian women. Int J Gynecol Obstet.

[54] Fahmy HH, Abd El-Rahman SI (2008). Determinants and health consequences of domestic violence among women in reproductive age at Zagazig District, Egypt. J Egypt Public Health Assoc.

[55] Guimei M, Fikry FE, Esheiba OM (2012). Patterns of violence against women in three communities in Alexandria, Egypt. MCN Am J Matern Child Nurs.

[56] Ibrahim ZM, Sayed Ahmed WA, El-Hamid SA, Hagras AM (2015). Intimate partner violence among Egyptian pregnant women: incidence, risk factors, and adverse maternal and fetal outcomes. Clin Exp Obstet Gynecol.

[57] Mamdouh HM, Ismail HM, Kharboush IF, Tawfik MM, El Sharkawy OG, Abdel-Baky M, Sallam HN (2012). Prevalence and risk factors for spousal violence among women attending health care centres in Alexandria, Egypt. East Mediterr Health J.

[58] Sayed AM, Elaziz KMA, Al HA, Abouseif IMB (2014). Domestic violence against Women attending primary health care units in Cairo: a cross sectional study. The Egyptian Journal of Community Medicine.

[59] Ahmed HM, Alalaf SK, Al-Tawil NG (2012). Screening for postpartum depression using Kurdish version of Edinburgh postnatal depression scale. Arch Gynecol Obstet.

[60] Al-Tawil NG (2012). Association of violence against women with religion and culture in Erbil Iraq: a cross-sectional study. BMC Public Health.

[61] Al-Atrushi HH, Al-Tawil NG, Shabila NP, Al-Hadithi TS (2013). Intimate partner violence against women in the Erbil city of the Kurdistan region, Iraq. BMC Womens Health.

[62] Al-Modallal H, Abuidhail J, Sowan A, Al-Rawashdeh A (2010). Determinants of depressive symptoms in Jordanian working women. J Psychiatr Ment Health Nurs.

[63] Al-Modallal H, Sowan AK, Hamaideh S, Peden AR, Al-Omari H, Al-Rawashdeh AB (2012). Psychological outcomes of intimate partner violence experienced by Jordanian working women. Health Care Women Int.

[64] Al-Modallal H (2012). Psychological partner violence and women's vulnerability to depression, stress, and anxiety. Int J Ment Health Nurs.

[65] Al-Modallal H (2012). Patterns of coping with partner violence: experiences of refugee women in Jordan. Public Health Nurs.

[66] Al-Modallal H, Abu Zayed I, Abujilban S, Shehab T, Atoum M (2015). Prevalence of intimate partner violence among women visiting health care centers in Palestine refugee camps in Jordan. Health Care Women Int.

[67] Al-Modallal H, Hamaideh S, Mudallal R (2014). Mental health status of Women in Jordan: a comparative study between attendees of governmental and UN relief and works Agency's health care centers. Issues Ment Health Nurs.

[68] Al-Natour A, Gillespie GL, Wang LL, Felblinger D (2014). A comparison of intimate partner violence between Jordanian nurses and Jordanian women. J Forensic Nurs.

[69] Al-Nsour M, Khawaja M, Al-Kayyali G (2009). Domestic violence against Women in Jordan: evidence from health clinics. J Fam Violence.

[70] Clark CJ, Silverman J, Khalaf IA, Abu Ra'ad B (2008). Abu Al Sha'ar Z, Abu Al Ata a, Batieha a: intimate partner violence and interference with women's efforts to avoid pregnancy in Jordan. Stud Fam Plan.

[71] Clark CJ, Bloom DE, Hill AG, Silverman JG (2009). Prevalence estimate of intimate partner violence in Jordan. East Mediterr Health J.

[72] Clark CJ, Silverman JG, Shahrouri M, Everson-Rose S, Groce N (2010). The role of the extended family in women's risk of intimate partner violence in Jordan. Soc Sci Med.

[73] Clark CJ, Shahrouri M, Halasa L, Khalaf I, Spencer R, Everson-Rose S (2012). A mixed methods study of participant reaction to domestic violence research in Jordan. J Interpers Violence.

[74] Clark CJ, Hill A, Jabbar K, Silverman JG (2009). Violence during pregnancy in Jordan: its prevalence and associated risk and protective factors. Violence Against Women.

[75] Okour AM, Badarneh R (2011). Spousal violence against pregnant women from a Bedouin community in Jordan. J Women's Health.

[76] Oweis A, Gharaibeh M, Alhourani R (2010). Prevalence of violence during pregnancy: findings from a Jordanian survey. Matern Child Health J.

[77] Awwad J, Ghazeeri G, Nassar AH, Bazi T, Fakih A, Fares F, Seoud M (2014). Intimate partner violence in a Lebanese population attending gynecologic care: a cultural perspective. J Interpers Violence.

[78] Hammoury N, Khawaja M (2007). Screening for domestic violence during pregnancy in an antenatal clinic in Lebanon. Eur J Pub Health.

[79] Hammoury N, Khawaja M, Mahfoud Z, Afifi RA, Madi H (2009). Domestic violence against women during pregnancy: the case of Palestinian refugees attending an antenatal clinic in Lebanon. J Women's Health.

[80] Khawaja M, Hammoury N (2008). Coerced sexual intercourse within marriage: a clinic-based study of pregnant Palestinian refugees in Lebanon. J Midwifery Womens Health.

[81] Usta J, Farver JAM, Pashayan N (2007). Domestic violence: the Lebanese experience. Public Health.

[82] Manoudi F, Chagh R, Es-soussi M, Asri F, Tazi I (2013). Family violence. Encephale.

[83] Boufettal H, Obaid B, Belhouss A, Hermas S, Noun M, Samouh N (2012). Physical violence during pregnancy in Morocco. J Gynecol Obstet Biol Reprod (Paris).

[84] Afifi ZE, Al-Muhaideb NS, Hadish NF, Ismail FI, Al-Qeamy FM (2011). Domestic violence and its impact on married women's health in eastern Saudi Arabia. Saudi Med J.

[85] Al-Faris H, Al-Faris H, Al-Faris E, Naghma N, Jamal A, AlQuaiz AM, Al-Thebaity R, Al-Zahrani M, Qusti N, Al-Ahmadi R (2013). A history of childhood maltreatment among spouses predicts violence against women. Ann Saudi Med.

[86] Eldoseri HM, Tufts KA, Zhang Q, Fish JN (2014). Adverse health effects of spousal violence among women attending Saudi Arabian primary health-care clinics. East Mediterr Health J.

[87] Fageeh Wafa M K (2014). Factors associated with domestic violence: a cross-sectional survey among women in Jeddah, Saudi Arabia. BMJ Open.

[88] Rachana C, Suraiya K, Hisham AS, Abdulaziz AM, Hai A (2002). Prevalence and complications of physical violence during pregnancy. Eur J Obstet Gynecol Reprod Biol.

[89] Tashkandi A, Rasheed FP (2009). Wife abuse: a hidden problem. A study among Saudi women attending PHC centres. East Mediterr Health J.

[90] Ahmed AM, Elmardi AE (2005). A study of domestic violence among women attending a medical Centre in Sudan. East Mediterr Health J.

[91] Maziak W, Asfar T (2003). Physical abuse in low-income women in Aleppo, Syria. Health Care Women Int.

[92] Maziak W, Asfar T, Mzayek F, Fouad FM, Kilzieh N (2002). Socio-demographic correlates of psychiatric morbidity among low-income women in Aleppo, Syria. Soc Sci Med.

[93] Haj-Yahia MM (2002). The impact of wife abuse on marital relations as revealed by the second Palestinian National Survey on violence against Women. J Fam Psychol.

[94] Reichenheim ME, Moraes CL (2004). Comparison between the abuse assessment screen and the revised conflict tactics scales for measuring physical violence during pregnancy. J Epidemiol Community Health.

[95] Devries KM, Kishor S, Johnson H, Stockl H, Bacchus LJ, Garcia-Moreno C, Watts C (2010). Intimate partner violence during pregnancy: analysis of prevalence data from 19 countries. Reprod Health Matters.

[96] Jewkes R (2010). Emotional abuse: a neglected dimension of partner violence. Lancet.

[97] Douki S, Nacef F, Belhadj A, Bouasker A, Ghachem R (2003). Violence against women in Arab and Islamic countries. Arch Womens Ment Health.

[98] Jansen HAFM, Watts C, Ellsberg M, Heise L, García-Moreno C (2004). Interviewer training in the WHO multi-country study on Women's health and domestic violence. Violence Against Women.

