# Supplementary material for: Prevalence of intimate partner violence against women in the Arab world: a systematic review
Source: BMC Int Health Hum Rights. 2019 Oct 22;19:29. doi: 10.1186/s12914-019-0215-5 (PMC6805386; doi:10.1186/s12914-019-0215-5)
Supplement: Supplementary file 1 — Operational definitions of physical, sexual, and/or emotional IPV in peer reviewed articles from the journal literature from Arab countries, 2000–2016. (DOCX 30 kb) [file 12914_2019_215_MOESM1_ESM.docx]

**Additional file 1.** Operational definitions of physical, sexual, and/or emotional IPV in peer reviewed articles from the journal literature from Arab countries, 2000-2016

| **Country** | **Lead author, year published** | **Perpetrator** | **Forms of violence (detailed operational definitions)** |
| --- | --- | --- | --- |
| **Egypt** | Diop Sidibe 2006 | Husband | ***Physical*** (Beaten) |
|  | Habib 2011 | Husband | ***Physical*** (hit, bit, slapped, pushed, punched, burned); ***sexual*** (denied sex, coerced sex, hurt during sex); ***emotional*** (frightening/angry look, insulted, abusive language, threatened with an object) – adapted from WHO |
|  | Seedhom 2012 | Husband | ***Physical*** (slapped, threw things, pushed, shoved, hit, kicked, dragged, beat, choked, burned, threatened with or attacked with a weapon); ***sexual*** (sexual intercourse against her will, physically forced sexual intercourse, forced to do sexually degrading acts); ***emotional*** (insulted, belittled, humiliated, scared on purpose, threats of violence)– based on WHO |
|  | Yount 2005 | Husband | ***Physical*** (beaten) – DHS module |
|  | Hassan 2004 | Husband | ***Physical*** (slapped, hit, punched, kicked, beaten); s***evere psychological*** (any of the following 3+ times: insulted, belittled, demeaned, verbally threatened her/someone close to her, raised hand to hit her without touching her, left her for 6+ months without financial support, unfaithful) |
|  | Ibrahim 2015 | Husband | ***Physical*** (slapped, pulled hair, bit, burned, punched, kicked, attacked with weapon or object);, ***sexual*** (forced to engage in sexual acts);, ***emotional*** (threatened with harm or abandonment; intimidated; humiliated; insulted or constantly criticized, accused or blamed; ignored, or ridiculed needs; controlled; withheld basic needs (food, shelter, medical care); deprived of liberty) |
|  | Yount 2014 | Current/most recent husband | ***Physical, sexual, emotional*** (not specified; based on DHS 2005 with additional items) |
| **Jordan** | Al-Badayneh 2012 | Husband | Not defined. Based on CTS2; included “exposure to some kind of violence against women or witnessing family violence”. |
|  | Khawaja 2005 | Husband | ***Physical (***slapped, grabbed, shoved, kicked, hit with fists, threw things, choked) |
|  | Shakhatreh 2005 | Not defined | “Domestic violence” (Not defined) |
| **Lebanon** | Khawaja 2004 | Husband | ***Physical*** (Beaten) |
| **Palestine** | Haj-Yahia 2000 | Husband | ***Physical*** (threw, kicked, broke something; pushed, kicked, tried to knock her over; pulled, slapped, threatened with knife, attacked with stick, belt, object, hands; choked; pulled hair, yanked clothes); ***sexual*** (tried to/had sex without consent; expressed dissatisfaction with sexual relationship in an insulting/degrading way); ***psychological*** (16 acts, e.g. insulted, yelled, intimidated, belittled, threatened, humiliated, prevented from visiting relatives/fiends; controlled behaviour/movements); ***economic*** (prevented from using money; misused family resources) |
|  |  |  |  |
|  | Clark 2010 | Husband | ***Physical*** (threw something, twisted arm, pulled hair, pushed, grabbed, slapped, attacked with weapon or sharp object, hit with object, strangled, burned); ***sexual*** (prevented from using contraceptives, physically forced or threatened to force unwanted sex); ***psychological*** (cursed, insulted, yelled, shouted, said things to provoke anger, called her fat, ugly, destroyed her things) |
| **Sudan** | Ali 2014 | Husband | ***Physical*** (slapped, threw things, pushed, shoved, hit, kicked, dragged, beat, choked, burned); ***sexua***l (coerced to perform sexual acts, physically forced sexual intercourse)***; psychologica***l (insulted, made to feel bad about herself, belittled, humiliated her in front of others, scared or intimidated, threatened to hurt her or someone she cared about); ***verbal insult*** (yelling, shouting, threatened with divorce and/or second marriage) |
| **Syria** | Tappis 2012 | Any household member | ***Physical, emotional, verbal*** (not specified) |
| **Egypt** | Bakr 2005 | Husband | ***Physical*** (threw objects, punched, ‘groped’, beat her or her children, forced sex); ***sexual*** (forced sex); ***control*** (shouted “for compliance”, put ambition down, put her down in front of people, left her short of money, stopped her from money, ‘took his own decision’, prevented her from visiting family or friends, limited her movement, locked her in house); ***threats*** (to harm or kill her, divorce her, take away her children; physically threats with arms, legs, objects, weapons) |
|  | Elnashar 2007 | Husband | ***Sexual*** (not defined) |
|  | Fahmy 2008 | Husband | ***Physical*** (slapped, threw something, hit during pregnancy); ***sexual*** (forced intercourse against her will); **psychological** (humiliated, belittled, threatened to divorce or expel her from home); ***social*** (prevented from seeing family or friends; took her money) |
|  | Guimei 2012 | Husband | ***Physical*** (beaten); ***sexual*** (forced sex) |
|  | Mamdouh 2012 | Current or most recent husband | ***Physical*** (slapped, beat, threw things, pushed. grasped, kicked, dragged, strangled, burned, threatened/attacked with weapon); ***sexual*** (forced sex against her will, when ill, during menses; hit or said hurtful words during sex, forced ‘insufferable’ things during sex); ***emotiona***l (insulted, ignored, treated indifferently, threatened to harm her or someone she cared about, divorce, expel her from home; insulted, humiliated, frightened her, forbade her from going out, social activities, visit parents); ***economic*** (makes her beg/borrow money, give him all her earnings; refuses to spend on home, spends her money on his pleasure; doesn’t work but forces her to work) |
|  | Sayed 2014 | Husband | ***Physical*** (threw something, punched, gripped, beat, beat children, forced sex); ***threats*** (threats, threats to divorce, take children; threats with a weapon, threats to kill her); ***control*** (shouted, “shattered” ambitions/hopes, embarrassed in front of others, prevented from having money, took decisions on his own, restricted from contacting family/friends, spied, locked in house.) |
| **Iraq** | Ahmed 2012 | Husband | ***Physical, sexual*** (not specified) |
|  | Al-Tawil 2012 | Husband | ***Physical*** (slapped, pushed, shook, threw something, twisted arm, pulled hair, hit with fist or object, kicked, dragged); ***sexual*** (physically forced to have sexual intercourse or perform any sexual act); ***psychological*** (insulted, scared/intimidated, humiliated, threatened to remarry, threatened with harm) - Adapted from WHO |
|  | Al-Atrushi 2013 | Husband | ***Physical*** (slapped, pushed, shook, threw things, twisted her arm or pulled her hair, hit, kicked, dragged, choked, burned, threatened or attacked with a weapon); ***sexual*** (physically forced, unwanted sexual intercourse; forced sex acts she considered degrading or humiliating); ***emotional*** (insulted, humiliated, threatened to divorce her, marry another woman, or hurt her; did something to scare her) - Adapted from WHO |
| **Jordan** | Al-Modallal 2010 | Current/former husband/fiancé | ***Work-related harassment*** (9 measures, e.g. bothered her at work; missed work due to abuse) |
|  | Al-Modallal 2012 | Current/former husband/fiancé | ***Physical, sexual*** (not specified; based on AAS); ***emotional*** (called names; threatened to or actually hurt somebody close to her; destroyed something of hers); ***controlling behaviours*** (limits her contact with family/friends, insists on knowing where she is, gets angry if she speaks to other men); ***economic*** (preventing from knowing about/accessing family income) |
|  | Al-Modallal 2014 | Current/former husband/fiancé | ***Physical, sexual, emotional*** (not specified; based on AAS) |
|  | Al-Natour 2014 | Husband | ***Physical, sexual, emotional*** (not specified; based on WAST tool) |
|  | Al-Nsour 2009 | Husband | ***Physical, emotional*** “abuse” (otherwise not defined) |
|  | Clark 2009 | Current/former husband | ***Physical*** (slapped, threw something, hit with fist or object; pushed, shoved, pulled hair; kicked, dragged, beat up, choked, burned, threatened/attacked with a weapon; ***sexual*** (physically forced unwanted sexual intercourse, forced sex act she found degrading or humiliating) ***psychological*** (blamed, insulted, made her feel bad, accused her of not fulfilling obligations to him/children, threatened to divorce, remarry; belittled, humiliated in front of others, intimidated, threatened to hurt her or someone she cared about); ***control*** (did not allow dialogue; disregarded her opinion; restricted her movement outside home, restricted visitors to home) - adapted from WHO |
|  | Okour 2011 | Husband | ***Physical*** (hit, threw things, threatened/attacked with weapon; kicked; pulled; pushed, pulled hair or clothes); ***sexual*** (forced intercourse against her will); ***psychological*** (insulted, made her feel bad, frightened her, ridiculed or humiliated her in front of others, controlled communication with family, prevented contact with friends, restricted her leaving the house) – adapted from WHO |
|  | Oweis 2010 | Husband | ***Physical, emotional, verbal, sexual*** Not defined. |
| **Lebanon** | Awwad 2014 | Husband | ***Physical*** (pushed, shoved, slapped, inflicted injury that required medical attention, threatened or attacked with a weapon or other object); ***sexual*** (forced vaginal or anal sex against her will; forced sexual practices that made her uncomfortable, like touching genital areas or breasts or making her touch genital organs against her will); ***verbal*** (insults or degrading remarks); ***emotional*** (repeatedly made fun in public or private; afraid, intimidated); ***social isolation*** (prevented from seeing friends/family); ***economic*** (denied access to money) |
|  | Hammoury 2007 | Household member | ***Physical*** (hit, slapped, kicked or physically hurt); ***sexual*** (forced sexual intercourse by husband); ***emotional*** (afraid of husband/someone in household) – based on AAS |
|  | Usta 2007 | Husband/other family member | ***Physical*** (hit, hurt); ***psychological*** (insulted, humiliated, threatened with harm); ***economic*** (denied financial support); ***emotional*** (threatened to divorce, separate, take children) – modified AAS |
| **Morocco** | Manoudi 2013 | Husband | ***Physical*** (beaten) |
|  | Boufettal 2012 | Partner | ***Physical*** (pushed, hit with hand/hard object, bitten, kicked, dragged, attacked with a knife, choked, burned); ***sexual*** (forced to have sex); sexual acts included in rates of physical abuse. |
| **Saudi Arabia** | Afifi 2011 | Any family member | ***Physical*** (slapped on face; threw something that could hurt; pushed, hit with a fist or something that could hurt; kicked, dragged, or beat up; choked, burned; threatened or attacked with a weapon); ***sexual*** (physically forced sex, had sex because of fear, forced to do something sexual she found degrading or humiliating); ***emotional*** (insulted, belittled, intimidated, threatened to harm her or someone she cared about, neglected) - Adapted from WHO |
|  | Al-Faris 2013 | Husband | ***Physical* (**slapped, threw something; twisted arm, shoved, pushed, hit with fists or object, kicked dragged, beaten up, choked, burned, threatened/attacked with weapon) - Adapted from WHO |
|  | Eldoseri 2014 | Husband | ***Physical*** (slapped, threw something that could hurt, shoved, pushed, hit with fist/object, kicked, dragged, beaten, choked, burned, threatened/attacked with weapon) - Adapted from WHO |
|  | Fageeh 2014 | Husband/other family member | ***Physical*** (pushed, beat, slapped, kicked, hit with a fist or object, pulled hair, dragged, burned; threatened or attacked with knife or gun); ***psychological*** (threatened, prevented from visiting or calling family members or friends, insulted); ***sexual*** (forced to have unwanted intercourse) |
|  | Rachana 2002 | Husband/in laws | ***Physical*** (physically hurt by husband; involved in physical fight) |
|  | Tashkandi 2009 | Husband | ***Physical*** (pushed, shoved, slapped, twisted arm, pulled hair, threw something that could hurt, slammed against wall, kicked, hit by something that could hurt, choked, punched, burnt); ***emotional*** (threatened to take another wife or divorce her; shouted, called ugly names, threatened with an object or weapon, destroyed personal belongings, spat on) |
| **Sudan** | Ahmed 2005 | Husband | ***Physical*** (pushed, kicked, threw objects, shoved, beat, physically hurt); ***threats*** (threatened physical injury); ***controlling behaviours*** (shouted, screamed, restricted her social life, checked her movements, etc.) |
| **Syria** | Maziak 2002 | Husband/other family member | ***Current physical*** (‘battering’ at least 3 times in past year and at least once in past 30 days); ***regular [physical] abuse*** (battering at least once weekly) |

**Notes:** NA: not available; WAST: Woman abuse screening tool; AAS: Abuse Assessment Screen; WHO: World Health Organization.
